# Nomogram for Prediction of Postoperative Delirium after Deep Brain Stimulation of Subthalamic Nucleus in Parkinson's Disease under General Anesthesia

**DOI:** 10.1155/2022/6915627

**Published:** 2022-11-29

**Authors:** Yu-Ting Ling, Qian-Qian Guo, Si-Min Wang, Li-Nan Zhang, Jin-Hua Chen, Yi Liu, Ruo-Heng Xuan, Bo Qu, Li-Ge Liu, Zhi-Shuang Wen, Jia-Kun Xu, Lu-Lu Jiang, Wen-Biao Xian, Bin Wu, Chang-Ming Zhang, Ling Chen, Jin-Long Liu, Nan Jiang

**Affiliations:** ^1^Department of Anesthesiology, First Affiliated Hospital of Sun Yat-sen University, Guangzhou 510080, Guangdong, China; ^2^Department of Neurology, First Affiliated Hospital of Sun Yat-sen University, Guangzhou 510080, Guangdong, China; ^3^Department of Neurosurgery, First Affiliated Hospital of Sun Yat-sen University, Guangzhou 510080, Guangdong, China

## Abstract

**Introduction:**

Postoperative delirium can increase cognitive impairment and mortality in patients with Parkinson's disease. The purpose of this study was to develop and internally validate a clinical prediction model of delirium after deep brain stimulation of the subthalamic nucleus in Parkinson's disease under general anesthesia.

**Methods:**

We conducted a retrospective observational cohort study on the data of 240 patients with Parkinson's disease who underwent deep brain stimulation of the subthalamic nucleus under general anesthesia. Demographic characteristics, clinical evaluation, imaging data, laboratory data, and surgical anesthesia information were collected. Multivariate logistic regression was used to develop the prediction model for postoperative delirium.

**Results:**

A total of 159 patients were included in the cohort, of which 38 (23.90%) had postoperative delirium. Smoking (OR 4.51, 95% CI 1.56–13.02, *p* < 0.01) was the most important risk factor; other independent predictors were orthostatic hypotension (OR 3.42, 95% CI 0.90–13.06, *p*=0.07), inhibitors of type-B monoamine oxidase (OR 3.07, 95% CI 1.17–8.04, *p*=0.02), preoperative MRI with silent brain ischemia or infarction (OR 2.36, 95% CI 0.90–6.14, *p*=0.08), Hamilton anxiety scale score (OR 2.12, 95% CI 1.28–3.50, *p* < 0.01), and apolipoprotein E level in plasma (OR 1.48, 95% CI 0.95–2.29, *p*=0.08). The area under the receiver operating characteristic curve (AUC) was 0.76 (95% CI 0.66–0.86). A nomogram was established and showed good calibration and clinical predictive capacity. After bootstrap for internal verification, the AUC was 0.74 (95% CI 0.66–0.83).

**Conclusion:**

This study provides evidence for the independent inducing factors of delirium after deep brain stimulation of the subthalamic nucleus in Parkinson's disease under general anesthesia. By predicting the development of delirium, our model may identify high-risk groups that can benefit from early or preventive intervention.

## 1. Introduction

Parkinson's disease (PD) is a common neurodegenerative disease in middle-aged and elderly individuals [1]. Deep brain stimulation (DBS) of the subthalamic nucleus (STN) is an effective method for the treatment of PD. Postoperative delirium is one of the common complications after DBS, with an incidence of 5.8%–40% [2–4]. It refers to an acute brain dysfunction mainly caused by the reduction of consciousness, cognitive impairment, and sensory disturbance due to various factors after operation [5, 6]. Postoperative delirium can aggravate patients' cognitive dysfunction, prolong the length of stay and total length of stay in the intensive care unit and ward, increase readmission rates and mortality, seriously reduce the quality of life after discharge, and bring heavy mental and economic burden to medical staff, family members, and society [6–8]. Thus, active prevention of postoperative delirium in PD patients is essential in the perioperative period of DBS. Early identification of risk factors for postoperative delirium in PD patients and active prevention and treatment measures can reduce the incidence of delirium [9]. Therefore, an effective clinical risk prediction model, which makes full use of intraoperative and preoperative risk factors rather than postoperative risk factors, may provide clinicians with more opportunities for the timely prevention of postoperative delirium.

There have been studies on the risk factors of delirium after DBS, but they were limited to awake DBS surgery and have not considered general anesthesia [4, 10, 11]. Since there are no unified standards for anesthesia for DBS surgery, different centers use variable anesthesia methods such as local anesthesia, sedation, intraoperative awakening, or whole course general anesthesia [12–15]. In the past, considering that narcotic drugs may interfere with the recognition and positioning of intraoperative electrophysiological signals and affect the outcomes, most centers kept patients awake to cooperate with the operation [16–18]. As the safety and feasibility of whole course general anesthesia are gradually confirmed, an increasing number of centers use general anesthesia to complete DBS surgery to provide patients with higher comfort and safety [19, 20]. However, anesthesia itself may be an independent risk factor for postoperative delirium [21]. For clinicians, especially anesthesiologists, there is an urgent need for a risk factor prediction model for postoperative delirium after DBS under whole course general anesthesia. The purpose of this study was to develop and internally verify a prediction model for postoperative delirium of STN-DBS under whole course general anesthesia, to accurately evaluate and identify high-risk groups, carry out active multidisciplinary interventions, prevent and reduce the occurrence of postoperative delirium, and promote postoperative recovery.

## 2. Methods

### 2.1. Patient Population

**Figure 1:**
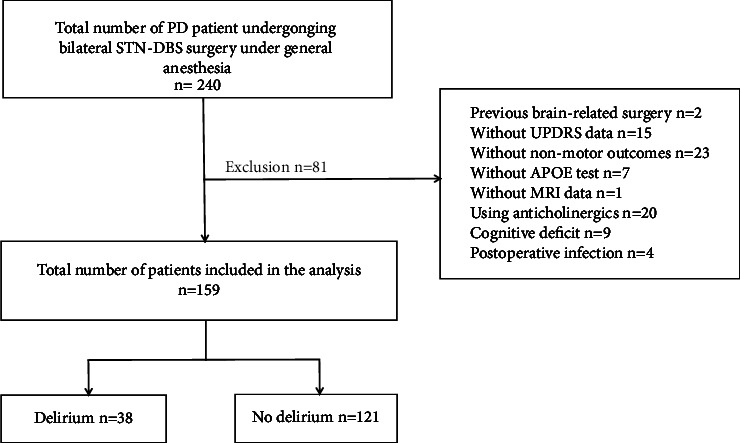
Flowchart detailing the selection of patients included in the retrospective analysis. PD, Parkinson's disease; STN, subthalamic nucleus; DBS, deep brain stimulation; MDS-UPDRS III, part 3 motor examination of the Movement Disorder Society Unified Parkinson's Disease Rating Scale; APOE, apolipoprotein E; MRI, magnetic resonance imaging.

The selected subjects were 240 patients with PD who underwent DBS of bilateral STN under general anesthesia in the First Affiliated Hospital of Sun Yat-sen University from June 2016 to November 2021. The exclusion criteria were age <18 years, a history of brain surgery, using anticholinergics, cognitive deficit, and postoperative infection. Individuals with some missing data or incomplete evaluation results were also excluded. Among them, there were 15 patients without a motor score, 23 without nonmotor outcomes, 7 patients without apolipoprotein E (APOE) detection in plasma, one patient had a cardiac pacemaker incompatible with magnetic resonance imaging (MRI) and thus had no MRI data, two patients had undergone brain surgery before surgery, 20 patients using anticholinergics, nine patients with abnormal mini-mental state examination (MMSE) scores, two patients had incision-induced infections, and two patients developed pneumonia. Therefore, a total of 159 cases were included in this study.  Figure 1 shows the flowchart of the study. The study protocol was approved by the local ethics committee (number 2021696) and was in accordance with the Declaration of Helsinki (DoH). Because this study was retrospective, the requirement of informed consent was waived.

### 2.2. Operations and Anesthesia Processes

Preoperative localization and surgical procedures were the same as those in previous studies [14]. The head was scanned with a 3-T MRI (Signa Excite; GE Medical Systems, Milwaukee, WI, USA) to identify and locate STN. MRI images were transmitted to the neuronavigation workstation through the image archiving and communication system. All antiparkinsonian medications were withdrawn for at least 12 h before the surgery. On the day of operation, the head frame was installed under local anesthesia and sent to the computed tomography room for scanning. The computed tomography image was fused with the preoperative MRI image to calculate the coordinates; the bilateral *x*, *y*, *z*, arc, and ring coordinates, as well as the planned electrode needle path, were obtained.

After entering the room, each patient's vital signs (including invasive arterial blood pressure, heart rate, electrocardiogram, and pulse oxygen saturation) and depth of anesthesia (Narcotrend, MonitorTechnik, Bad Bramstedt, Germany, or SedLine monitor, Masimo Corporation, Irvine, CA, USA) were monitored. Anesthesia was induced as follows: propofol target-controlled infusion (TCI, 3-4 *μ*g/mL) or etomidate (0.3 mg/kg i.v. or TCI 0.3 *μ*g/mL), remifentanil (TCI 4 ng/mL) or sufentanil (0.2 *μ*g/kg, i.v.), and cis-atracurium (0.2 mg/kg, i.v.). After the loss of patient's consciousness, endotracheal intubation was performed under laryngoscopy. Then, the ventilator was connected to maintain good ventilation. Maintenance of anesthesia was achieved as follows: propofol TCI (1–4 *μ*g/mL) or etomidate (TCI 0.1–0.5 *μ*g/mL or 1–6 *μ*g/kg/min), remifentanil TCI (2–4 ng/mL), and cis-atracurium (1.5 *μ*g/kg/min). All of the patients received local scalp anesthesia with ropivacaine 0.5%. Microelectrode recording was performed to verify the correct location of the target coordinates. Permanent quadripolar DBS electrodes were implanted along the optimal trajectory after *X*-ray examination. The pulse generator and extension lead were implanted under anesthesia with propofol, remifentanil, and sufentanil. During the operation, a small dose of norepinephrine (0.01–0.1 *μ*g/kg/min) was used to maintain the mean arterial blood pressure above 80 mmHg. For adjuvant medication, palonosetron 0.25 mg, flurbiprofen 1 mg/kg, and dexmedetomidine 0.3 *μ*g/kg were used. After the operation, the patients were transferred to an intensive care unit. The pulse generator was started after one month.

### 2.3. Data Collection

We collected data on 40 candidate risk factors for postoperative delirium, including age, gender, body mass index (BMI), comorbidity (hypertension, diabetes, and heart disease), smoking history, surgical history, delirium history, and drug-induced hallucination history. The clinical characteristics of PD included the duration of PD, age of onset, duration of antiparkinsonian medication, onset limb, and disease severity (Hoehn and Yahr stages, off and on). The preoperative evaluation was completed one week in advance by a designated physician trained in neurology,and included motor evaluation (improvement rate of the levodopa challenge test, motor classification, motor score (Movement Disorder Society Unified Parkinson's Disease Rating Scale, part III off score), and nonmotor evaluation (involving cognition, anxiety, depression, daytime sleepiness, autonomic nerve dysfunction evaluation (orthostatic hypotension), and sleep disorder evaluation (rapid eye movement sleep behavior disorder, RBD))). The nonmotor evaluation scales used were the MMSE, Hamilton anxiety scale (HAMA), Hamilton depression scale (HAMD), and Epworth sleepiness scale (ESS). Cerebral atrophy, silent brain ischemia, and infarction (such as scattered frontal and parietal ischemia or silent lacunar infarction) on preoperative MRI, preoperative APOE levels in plasma, total protein, and albumin levels were also recorded. The preoperative medication was also recorded, including levodopa equivalent daily dose [22], antiparkinsonian medications (amantadine/type-B monoamine oxidase inhibitors (MAOBIs)/catechol-O-methyl transferase (COMT) inhibitors), antipsychotics, and hypnotics. Intraoperative blood pressure (intraoperative mean arterial pressure and systolic arterial pressure), the occurrence of intraoperative hypotension, anesthesia time, use of dexamethasone, and types of sedative anesthetics used (propofol or etomidate) were obtained through electronic medical records of surgery and anesthesia. Orthostatic hypotension was defined as a drop in arterial systolic pressure and diastolic pressure between the lying and upright positions of more than 20 mmHg and 10 mmHg within 3 min, respectively [23]. The diagnosis of brain atrophy, silent brain ischemia, and infarction on preoperative MRI was obtained from the official report of imaging examination. Patients with silent cerebral ischemia and infarction indicated on preoperative MRI denied having cerebrovascular disease. Intraoperative hypotension was defined as systolic arterial pressure <70 mmHg for 5 min or more, mean arterial pressure <49 mmHg for 5 min or more, and diastolic pressure <30 mmHg for 5 min or more [24]. Hypoproteinemia was defined as serum total protein levels below 60 g/L or albumin levels below 35 g/L. Among the 159 patients, the postoperative adverse events within 7 days related to surgery, anesthesia, and other complications (such as hiccups) were recorded. The confusion assessment method (CAM) was used for the evaluation of delirium [25]. Delirium was assessed from the first postoperative day of discharge and confirmed by daily nursing as well as family and accompanying personnel interviews.

### 2.4. Statistical Analysis

Stata version 16.0 software (STATA Corp., College Station, Texas, USA) and SPSS version 26.0 software (IBM Corp, Armonk, NY, USA) were used for all statistical analyses. Descriptive analysis was used to summarize the baseline characteristics of the patients. Quantitative data are expressed as the mean ± standard deviation, and qualitative data are expressed as the number of cases and percentage. A univariate logistic regression analysis was used to determine the association between each candidate risk factor and delirium. The criterion of *p* < 0.05 was used to screen the candidate predictors. The variables related to delirium in the univariate analysis were included in the binary logistic regression analysis to establish the prediction model of postoperative delirium. To decide which model was better, the stepwise backward/forward/backward-forward analysis and the Akaike information criterion (AIC) generated by each of them were considered. To assess the discrimination performance of the models, the area under the receiver operating characteristic curve (AUC) was calculated. The range of the AUC was 0-1. If the AUC was greater than 0.7, the model was considered to have good performance. A nomogram was constructed to assist in clinical applications. The Hosmer–Lemeshow test and calibration curve were performed to evaluate the calibration of the model. The decision curve analysis (DCA) was applied to assess the nomogram's clinical utility. The internal validation of the model used the bootstrap method of 1000 repeated sampling steps.

## 3. Results

### 3.1. Population Characteristics

The number of cases included in this study was 159. The average age was 59.65 ± 8.83 years, and there were 106 men (66.67%). The average body mass index (BMI) was 23.02 ± 3.30 kg/m^2^; the average age at onset was 50.31 ± 8.86 years; the average duration of PD was 9.67 ± 3.93 years; the average duration of antiparkinsonian medication was 8.61 ± 3.59 years; the average Hoehn and Yahr stage, the off-stage was 3.13 ± 0.71, and the on-stage was 2.14 ± 0.66; the average result of the levodopa challenge test was 62.11 ± 13.13% (Table 1). Among the 159 patients, no one experienced ischemia, bleeding, decompensation of previous diseases, metabolic or hydroelectrolytic disorders, or death. Interestingly, a seven-day follow-up of adverse events showed that 22 cases (13.84%) developed transient hiccups. There were 38 cases of postoperative delirium (incidence of 23.90%).

### 3.2. Univariate and Multivariate Analyses

Of the initial candidate variables, 10 were retained in the final multivariate model (Table 2). These variables were as follows: smoking, cardiac disease, drug-induced hallucination history, HAMA score, orthostatic hypotension, plasma APOE level, preoperative silent brain infarction or ischemia, MAOBIs, use of etomidate, and use of dexamethasone. Based on the lowest AIC value of 156.99, we used the stepwise backward-forward analysis logistic regression model to analyze the above factors. The multivariate logistic regression analysis showed that the risk factors for postoperative delirium were smoking (OR 4.51, 95% CI 1.56–13.02, *p* < 0.01), orthostatic hypotension (OR 3.42, 95% CI 0.90–13.06, *p*=0.07), preoperative MRI with silent brain ischemia or infarction (OR 2.36, 95% CI 0.90–6.14, *p*=0.08), MAOBIs (OR 3.07, 95% CI 1.17–8.04, *p*=0.02), HAMA score (OR 2.12, 95% CI 1.28–3.50, *p* < 0.01), and APOE level in plasma (OR 1.48, 95% CI 0.95–2.29, *p*=0.08).

### 3.3. Model Discrimination, Calibration, Clinical Utility Evaluation, and Verification

**Figure 2:**
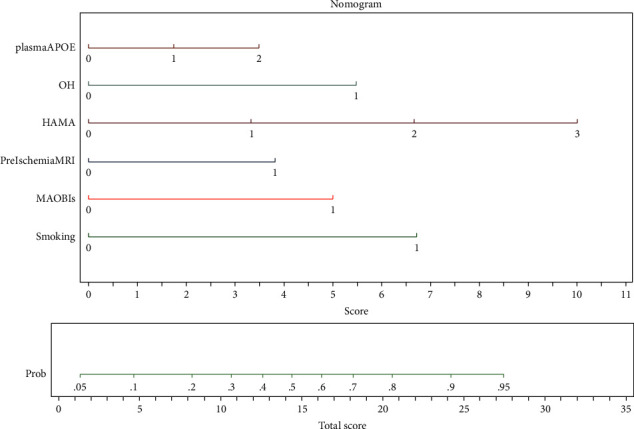
Nomogram to predict the risk of postoperative delirium. HAMA, Hamilton anxiety scale; MAOBIs, type-B monoamine oxidase inhibitors; OH, orthostatic hypotension; APOE: apolipoprotein E; PreIschemiaMRI, silent brain ischemia and cerebral infarction in preoperative magnetic resonance imaging.

**Figure 3:**
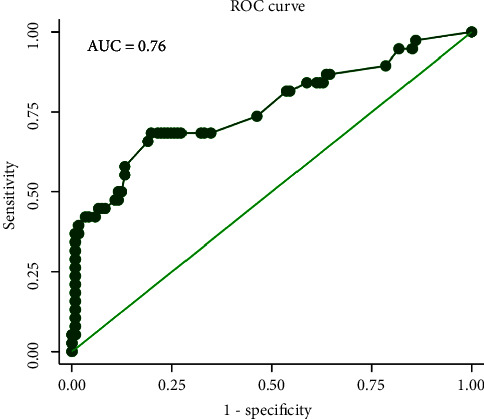
Area under the receiver operating characteristic (ROC) curve (AUC) plots for the predictive model of delirium after deep brain stimulation of the subthalamic nucleus in Parkinson's disease under general anesthesia.

**Figure 4:**
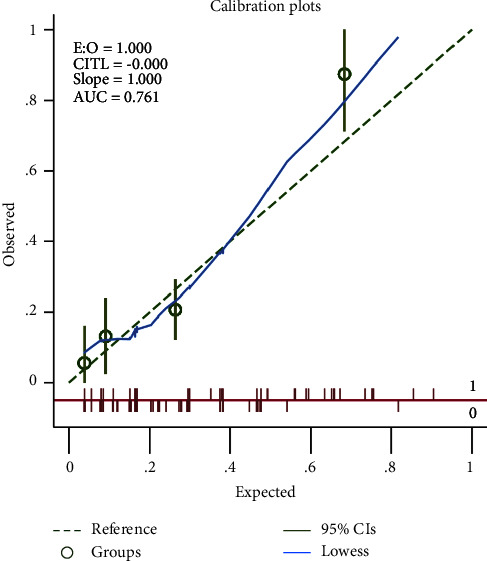
Calibration plot for predicting postoperative delirium probability.

**Figure 5:**
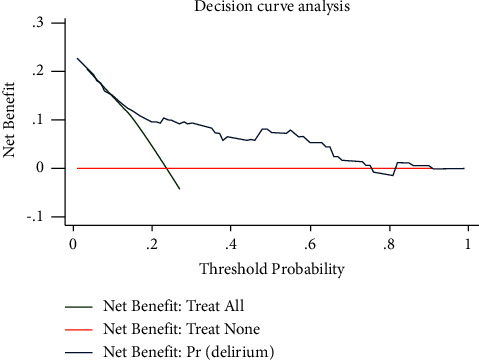
Decision curve analysis (DCA) of the nomogram.

**Figure 6:**
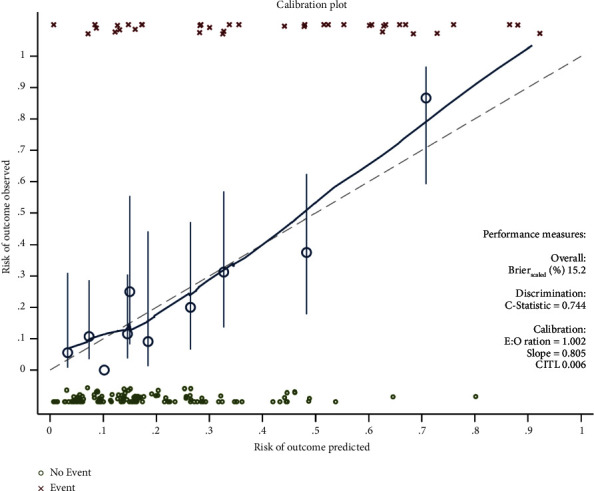
Calibration plot for predicting the postoperative delirium model derived using 1000 bootstrap iterations.

Six valuable factors (smoking, orthostatic hypotension, preoperative MRI with silent brain ischemia or infarction, MAOBIs, HAMA score, and APOE level in plasma) were selected to establish the predictive model. Then, a nomogram for prediction of postoperative delirium after DBS of STN in PD under general anesthesia was built on the basis of a multivariate logistic regression model (Figure 2). The nomogram achieved good discriminatory performance, as reflected by an AUC of 0.76 (95% CI 0.66–0.86) (Figure 3). Moreover, the nomogram showed good calibration (Figure 4), with a nonsignificant difference (chi-square = 13.08, *p*=0.16) derived from the Hosmer–Lemeshow test. The DCA curve revealed good clinical utility. As shown in Figure 5, if the threshold probability is 0.25, patients would benefit more from using this nomogram than treating either all or none in this study. The final model was internally validated by bootstrap resampling. After 1000 bootstrap replications, the model still showed good accuracy, with an AUC of 0.74 (95% CI 0.66–0.83, Figure 6).

## 4. Discussion

Delirium is common in patients with PD and can occur in outpatient/inpatient settings and after surgery [6]. The general anesthesia has become an option for DBS anesthesia in recent years because of its sufficient comfort and safety. To the best of our knowledge, there have been no studies on the incidence, related factors, and nomogram of postoperative delirium in PD after DBS under general anesthesia. The advantage of this study is that the candidate risk factors involved a number of factors, such as baseline characteristics, clinical characteristics of PD, laboratory examination, imaging examination, and factors related to anesthesia. This is necessary for the study of postoperative delirium affected by multiple factors. Especially for anesthesia, some reports have indicated that opioid doses and intraoperative hypotension increase the incidence of postoperative delirium [26, 27]. However, in this study, the factors finally identified as the independent risk factors were not related to anesthesia. This may be due to the use of a unified and optimized anesthesia scheme to control confounding factors, such as a unified opioid dose and the concept of routine use of norepinephrine to avoid intraoperative hypotension. We found that smoking, orthostatic hypotension, preoperative MRI indicating silent brain ischemia or infarction, taking MAOBIs, abnormal HAMA score, and abnormal plasma APOE level were independent risk factors for delirium after DBS under general anesthesia in PD patients.

Our findings suggest that smoking is the strongest independent risk factor for delirium after DBS. Previous studies mainly focused on the relationship between smoking and cognitive impairment. Specifically, chronic cigarette smoking and withdrawal from smoking and nicotine use have a negative impact on cognitive function [28–32]. Only a few studies on cardiac, vascular, and other types of surgical outcomes have found that chronic cigarette smoking has an increased risk of developing postoperative delirium [33–35]. To our knowledge, no study has reported a correlation between smoking and delirium after DBS. The previous studies may not have collected such information. Some reviews have shown that smoking is considered to be a high-risk factor for delirium during hospitalization and in the ICU, but there is no special mention of PD patients [36]. The mechanism of delirium caused by smoking is also rarely studied. The possible mechanism is microvascular changes/increased atherosclerotic burden/withdrawal effects of nicotine [35–38].

Preoperative orthostatic hypotension was an independent risk factor for postoperative delirium in this study. The incidence of orthostatic hypotension in PD is as high as 20%–50%, and a considerable number of patients do not show obvious symptoms, so the appropriate treatment is delayed [39]. The relationship between orthostatic hypotension and postoperative delirium is not clear. Only one prospective cohort study has shown that delirium in the elderly may be related to abnormal autonomic nervous system responses to head-up tilt testing [40]. However, other studies have shown that orthostatic hypotension is related to cognitive impairment and can also increase the risk of dementia [41–43]. The possible mechanism is related to long-term, repeated cerebral hypoperfusion or age-related progressive impairment of the brain's ability to automatically regulate cerebral blood flow. While DBS surgery needs to avoid intracranial hemorrhage caused by high blood pressure, it is also necessary to avoid hypotension throughout the perioperative period, which needs to be considered. According to the results of our study, for PD patients with orthostatic hypotension, multidisciplinary treatment should be carried out to actively prevent hypotension (wearing elastic mesh stockings, adjusting antiparkinsonian drugs and other drugs that can cause hypotension, vasoactive drugs to control blood pressure to avoid low or high blood pressure, and monitoring of blood pressure level in the ICU when recovering antiparkinsonian drugs after DBS) [23]. In particular, intraoperative hypotension is associated with postoperative delirium [27], but in this study, due to the routine use of low-dose norepinephrine to maintain blood pressure during anesthesia, we had fewer patients with intraoperative hypotension. Therefore, we were unable to study the effect of intraoperative hypotension on postoperative delirium.

Preoperative MRI showed silent brain ischemia and cerebral infarction, which could predict the increased risk of postoperative delirium. This finding is similar to the results of previous studies on the prediction of postoperative delirium in DBS [10, 11]. Microembolization is considered an important factor leading to postoperative delirium, and there may be many mechanisms for its occurrence [44]. Cerebral embolism with cholesterol can change the permeability of the blood-brain barrier and activate microglia [45]. In addition, it may also be related to synaptic and neuronal dysfunction caused by proinflammatory cytokines.

MAOBIs are commonly used drugs for patients with advanced Parkinson's disease. These drugs can be started in the early stage of the disease, reducing the use of dopamine-related medications and reducing drug complications [46]. However, MAOBIs, especially selegiline, are considered to cause some psychiatric adverse effects, such as delirium, hallucinations, and agitation [47, 48]. DBS surgery usually requires patients to stop all antiparkinsonian drugs, including MAOBIs, for at least 12 hours before surgery. According to previous reports, MAOBIs can have drug interruption reactions, and the most common manifestation includes mental symptoms [49]. Although previous studies reported mental symptoms after DBS, many of them did not take MAOBIs into account. One study compared the effects of levodopa alone and selegiline combined with levodopa on autonomic nerve function in patients with PD [50]; it was found that the latter may be related to severe orthostatic hypotension. This suggests that there may be a collinear relationship between MAOBIs and orthostatic hypotension, the two risk factors of postoperative delirium as revealed in our study.

Our results also showed that higher HAMA scores were associated with a greater risk of postoperative delirium. HAMA is a scale that is commonly used in PD patients with anxiety disorder. It has good specificity and sensitivity [51]. Patients with preoperative anxiety disorder are more likely to have postoperative delirium, which has been studied in other surgeries and populations [52]. For DBS research, it is possible that DBS electrode stimulation to the limbic territories of the STN or current diffusion may cause an imbalance of related neurotransmitters in the substantia nigra [53]. The limbic territories have a certain impact on cognition and emotion. Therefore, it is particularly important to fully evaluate the patient's neuropsychiatric status and select the target before operation [54].

Plasma APOE levels were also shown to be a risk factor for postoperative delirium in this study. APOE is a glycoprotein that is mainly expressed in the brain, primarily by astrocytes and microglia [55]. Many studies have shown that the APOE *ε*4 genotype significantly correlates with postoperative delirium [56]. We did not perform genotyping because this was a retrospective study, but the results of APOE in plasma also suggested this correlation. Our results suggested that if APOE genotyping is not available, postoperative delirium can be predicted by simply measuring the level of total plasma APOE protein. Moreover, APOE, whether below or above the normal level, was related to the occurrence of postoperative delirium.

In this study, the use of dexamethasone was not a predictor in the multivariate analysis, although the anti-inflammatory effect of dexamethasone may have many potential beneficial effects on the prevention of postoperative delirium, including the possible reduction of brain edema and neuroinflammation [57]. However, the relationship between dexamethasone and postoperative delirium is still controversial. A small controlled study did show that postoperative dexamethasone administration was associated with a reduced incidence of insanity [58]. However, there are also randomized controlled, double-blind studies that have shown that intraoperative dexamethasone administration did not reduce the incidence and duration of psychosis in the first four days after cardiac surgery [59]. There is even a belief that dexamethasone increases the risk of insanity because insanity is a known complication of long-term and/or high-dose glucocorticoid treatment [60]. The postoperative serum cortisol concentration was higher in patients with delirium. In this study, the use of etomidate was also considered to be associated with postoperative delirium in the univariate analysis. Etomidate is also a drug that can affect patients' serum cortisol levels. It is well known that etomidate can inhibit the synthesis of adrenal steroids and significantly reduce the concentration of plasma cortisol [61]. A few previous studies have shown that etomidate is related to postoperative delirium [62, 63]. Recent studies have pointed out that etomidate is the only important variable associated with delirium after electroshock treatment [64]. Some people believe that etomidate may lead to the long-term reduction of postoperative cortisol levels, resulting in proinflammatory effects, which may be related to postoperative delirium [65]. However, etomidate was not an independent risk factor for postoperative delirium in this study. We speculate that etomidate may offset the effect of adrenocortical inhibition on postoperative delirium by maintaining hemodynamic stability. More importantly, the role of the hypothalamic-pituitary axis and serum cortisol in the pathogenesis of postoperative delirium is controversial [66–68].

This study has some limitations. First, because this study was retrospective, we were not able to provide data on debilitation and depth of anesthesia as candidate factors, although these factors are considered related to postoperative delirium [69, 70]. However, we believe that BMI can approximately replace the effect of debilitation. As for the depth of anesthesia, we used the depth of anesthesia monitor to guide the anesthetics for each patient and maintained the depth of anesthesia at the level of C to D. Therefore, lack of debilitation and depth of anesthesia information may have little effect on this study. Second, in this study, preoperative cognitive function screening by MMSE may not be enough to completely exclude patients with preoperative cognitive dysfunction. Therefore, the conclusions of this study are only applicable to PD patients with normal MMSE screening. In the future, cognitive function needs to be assessed by rigorous neuropsychological tests. This was a single-center study with a small sample size. The main reason is that DBS under whole course general anesthesia has not been widely used, and the study of delirium in elderly PD patients is quite time-consuming and laborious. Thus, our future research will try to verify our conclusions externally through multicenter and large sample studies.

## 5. Conclusions

Smoking, orthostatic hypotension, preoperative MRI indicating silent brain ischemia or infarction, taking MAOBIs, abnormal HAMA score, and abnormal plasma APOE levels are independent predictors of delirium development after STN-DBS under general anesthesia in patients with PD. We constructed a reliable and useful nomogram, which can accurately predict the development of delirium after DBS surgery under general anesthesia. The tool is easy to use and can help clinicians, especially anesthesiologists, in the treatment decision-making process. However, due to the limited number of factors included in this study and the single-center design, only internal validation was carried out. It is thus necessary to carry out a multicenter study with a larger sample size to further explore and verify the current research results.

## Figures and Tables

**Table 1:** Baseline characteristics and univariate analysis of postoperative delirium in PD patients after STN-DBS under general anesthesia.

	Total population	Nondelirium group	Delirium group	*P* value
	*n* = 159	*n* = 121	*n* = 38	
General information				
Age (years)	59.65 ± 8.83	59.17 ± 8.92	61.21 ± 8.44	0.21
> 65 years	51 (32.08)	36 (29.75)	15 (39.47)	0.27
Male sex	106 (66.67)	81 (66.94)	25 (65.79)	0.90
Body mass index (kg/m^2^)	23.02 ± 3.30	23.02 ± 3.34	23.03 ± 3.24	
Normal (18.5–23.9)	73 (45.91)	50 (41.32)	23 (60.53)	0.12
Low (<18.5)	20 (12.58)	17 (14.05)	3 (7.89)	0.07
High (>23.9)	66 (41.51)	54 (44.63)	12 (31.58)	0.74
Smoking (current/former)	24(15.09)	14(11.57)	10(26.32)	0.03
Hypertension	41 (25.79)	32 (26.45)	9 (23.68)	0.73
Diabetes	12 (7.55)	10 (8.26)	2 (5.26)	0.55
Cardiac disease	15 (9.43)	8 (6.61)	7 (18.42)	0.04
Surgical history	81 (50.94)	62 (51.24)	19 (50.00)	0.89
History of delirium	4 (2.52)	1 (0.83)	3 (7.89)	0.05
History of drug-induced hallucination	34 (21.38)	21 (17.36)	13 (34.21)	0.03
Age at onset of PD (years)	50.31 ± 8.86	49.63 ± 8.93	52.47 ± 8.40	0.18
≥60	22 (13.84)	15 (12.40)	7 (18.42)	
50–59	71 (44.65)	52 (42.98)	19 (50.00)	
40–49	46 (28.93)	38 (31.40)	8 (21.05)	
≤39	20 (12.58)	16 (13.22)	4 (10.53)	
Duration of PD over 10 (years)	69 (43.40)	54 (44.63)	15 (39.47)	0.58
First affected extremity				
Left side	73 (45.91)	56 (46.28)	17 (44.74)	0.90
Right side	76 (47.80)	58 (47.93)	18 (47.37)	0.64
Both sides or head or neck	10 (6.29)	7 (5.79)	3 (7.89)	0.66
Hoehn and Yahr stage (off)	3.13 ± 0.71	3.16 ± 0.71	3.04 ± 0.70	0.36
Hoehn and Yahr stage (on)	2.14 ± 0.66	2.15 ± 0.66	2.11 ± 0.65	0.75
Preoperative motor assessments				
Levodopa challenge test (%)	62.11 ± 13.13	62.94 ± 12.74	59.38 ± 14.19	0.41
MDS-UPDRS III >66, off	27 (16.98)	22 (18.18)	5 (13.16)	0.47
Clinical phenotypes of motor impairment				
Tremor domination	88 (55.35)	67 (55.37)	21 (55.26)	0.99
Postural/gait difficulty	58 (36.48)	44 (36.36)	14 (36.84)	0.95
Indeterminate	13 (8.18)	10 (8.26)	3 (7.89)	0.94
Preoperative nonmotor assessments				
MMSE score	27.70 ± 2.25	27.88 ± 2.19	27.11 ± 2.37	0.07
ESS score	7.42 ± 5.26	7.17 ± 4.97	8.21 ± 6.09	0.50
RBD	65 (40.88)	47 (38.84)	18 (47.37)	0.35
HAMA score	6.67 ± 5.78	5.89 ± 5.16	9.16 ± 6.91	<0.01
<8, normal	95 (59.75)	79 (65.29)	16 (42.11)	
8–14, anxiety, may be	46 (28.93)	32 (26.45)	14 (36.84)	
15–21, anxiety (mild)	14 (8.81)	8 (6.61)	6 (15.79)	
>21, anxiety (moderate/severe)	4 (2.52)	2 (1.65)	2 (5.26)	
HAMD score	8.84 ± 6.24	8.28 ± 5.83	10.61 ± 7.18	0.11
<8, normal	84 (52.83)	66 (54.55)	18 (47.37)	
8–20, suspicious depression	64 (40.25)	50 (41.32)	14 (36.84)	
>20, depression	11 (6.92)	5 (4.13)	6 (15.79)	
Orthostatic hypotension	13 (8.18)	6 (4.96)	7 (18.42)	0.01
Preoperative laboratory and MRI examination				
Hypoproteinemia	11 (6.92)	6 (4.96)	5 (13.16)	0.09
Plasma APOE level (mg/L)				
Normal (27–45)	93 (58.49)	77 (63.64)	16 (42.11)	0.04
Low (<27)	11 (6.92)	6 (4.96)	5 (13.16)	0.06
High (>45)	55 (34.59)	38 (31.40)	17 (44.74)	0.36
Brain atrophy	73 (45.91)	56 (46.28)	17 (44.74)	0.87
Silent brain infarction/ischemia	102 (64.15)	72 (59.50)	30 (78.95)	0.03
Preoperative medication				
LEDD > 1000 mg	38 (23.90)	28 (23.14)	10 (26.32)	0.69
Amantadine	22 (13.84)	14 (11.57)	8 (21.05)	0.15
COMT inhibitors	79 (49.69)	62 (51.24)	17 (44.74)	0.49
MAOBIs	31 (19.50)	18 (14.88)	13 (34.21)	0.01
Antipsychotics	19 (11.95)	14 (11.57)	5 (13.16)	0.79
Hypnotics	57 (35.85)	45 (37.19)	12 (31.58)	0.53
Anesthesia and surgery				
Anesthesia time > 420 min	63 (39.62)	44 (36.36)	19 (50.00)	0.14
Use of etomidate	32 (20.13)	19 (15.70)	13 (34.21)	0.02
Intraoperative hypotension	2 (1.26)	1 (0.83)	1 (2.63)	0.41
MAP (mmHg)	78.76 ± 6.70	78.21 ± 6.87	80.53 ± 5.83	0.07
SAP (mmHg)	114.94 ± 8.92	114.33 ± 8.56	116.90 ± 9.84	0.12
Use of dexamethasone	109 (68.55)	90 (74.38)	19 (50.00)	0.01

Numbers indicate means ± standard deviations (SD) or number (percentage). Differences between the nondelirium group and delirium group were analyzed using univariate logistic regression analysis. Significance was recognized when *p* < 0.05. PD, Parkinson's disease; STN, subthalamic nucleus; DBS, deep brain stimulation; MDS-UPDRS III, part 3 motor examination of the Movement Disorder Society Unified Parkinson's Disease Rating Scale; MMSE, mini-mental state examination; ESS, Epworth sleepiness scale; RBD, rapid eye movement sleep behavior disorder; HAMA, Hamilton anxiety scale; HAMD, Hamilton depression scale; MRI, magnetic resonance imaging; APOE, apolipoprotein E; LEDD, levodopa equivalent daily dose; COMT, catechol-O-methyl transferase; MAOBIs, type-B monoamine oxidase inhibitors; MAP, mean arterial pressure; SAP, systolic arterial pressure.

**Table 2:** Univariate and multivariate analyses of postoperative delirium in PD patients after STN-DBS under general anesthesia.

	Univariate analysis				Multivariate analysis			
	Odds ratio	Lower	Upper	*P*	Odds ratio	Lower	Upper	*P*
Smoker (current/former)	2.73	1.10	6.79	0.03	4.51	1.56	13.02	<0.01
Cardiac disease	3.19	1.07	9.48	0.04				
History of drug-induced hallucination	2.48	1.09	5.62	0.03				
HAMA score	1.87	1.19	2.95	<0.01	2.12	1.28	3.50	<0.01
Orthostatic hypotension	4.33	1.36	13.81	0.01	3.42	0.90	13.06	0.07
Plasma APOE level (mg/L)				0.04	1.48	0.95	2.29	0.08
Low (<27)	0.46	0.21	1.02	0.06				
High (>45)	1.86	0.50	6.96	0.36				
Silent brain infarction/ischemia	2.55	1.08	6.23	0.03	2.36	0.90	6.14	0.08
MAOBIs	2.98	1.29	6.87	0.01	3.07	1.17	8.04	0.02
Use of etomidate	2.79	1.22	6.40	0.02				
Use of dexamethasone	0.34	0.16	0.73	0.01				

Significance was recognized when *p* < 0.05. PD, Parkinson's disease; STN, subthalamic nucleus; DBS, deep brain stimulation; HAMA, Hamilton anxiety scale; APOE, apolipoprotein E; MAOBIs, type-B monoamine oxidase inhibitors.

## Data Availability

All data generated during and/or analyzed during the current study are available from the corresponding author upon request.
